# Medicinal Mushrooms in Metastatic Breast Cancer: What Is Their Therapeutic Potential as Adjuvant in Clinical Settings?

**DOI:** 10.3390/cimb46070450

**Published:** 2024-07-17

**Authors:** Fabrizio De Luca, Elisa Roda, Paola Rossi, Maria Grazia Bottone

**Affiliations:** 1 Department of Biology and Biotechnology “L. Spallanzani”, University of Pavia, 27100 Pavia, Italy; fabrizio.deluca@unipv.it (F.D.L.); paola.rossi@unipv.it (P.R.); 2 Laboratory of Clinical & Experimental Toxicology, Pavia Poison Centre, National Toxicology Information Centre, Toxicology Unit, Istituti Clinici Scientifici Maugeri, IRCCS Pavia, 27100 Pavia, Italy; elisa.roda@icsmaugeri.it

**Keywords:** metastatic breast cancer, medicinal mushrooms, micotherapy, *Lentinula edodes*, *Grifola frondosa*, *Ganoderma lucidum*, *Ophiocordyceps sinensis*, *Agaricus blazei*

## Abstract

Breast cancer (BC) is the most commonly diagnosed tumor, remaining one of the leading causes of morbidity and mortality in females worldwide, with the highest rates in Western countries. Among metastatic BC (MBC), triple-negative breast cancer (TNBC) is characterized by the lack of expression of specific receptors, and differs from other subgroups of BC for its increased growth and fast spreading, with reduced treatment possibilities and a worse outcome. Actually, MBC patients are extremely prone to metastasis and consequent relapses, which affect distant target organs (e.g., brain, lung, bone and liver). Hence, the comprehension of biological mechanisms underlying the BC metastatization process is a key requirement to conceive/set up innovative medicinal strategies, with the goal to achieve long-lasting therapeutic efficacy, reducing adverse effects, and also ameliorating Quality of Life (QoL). Bioactive metabolites isolated from medicinal mushrooms (MMs) used as a supportive treatment, combined with conventional oncology, have recently gained wide interest. In fact, mounting evidence has revealed their peculiar promising immunomodulatory, anti-inflammatory and anticancer activities, even though these effects have to be further clarified. Among the group of most promising MMs are *Lentinula edodes*, *Grifola frondosa*, *Ganoderma lucidum*, *Ophiocordyceps sinensis* and *Agaricus blazei*, which are already employed in conventional cancer protocols in Asia and China. Recently, a growing number of studies have focused on the pharmacology and feasibility of MM-derived bioactive compounds as a novel valuable approach to propose an effective adjuvant therapy for MBC patients’ management. In this review, we summarized the current state of knowledge on the abovementioned MM-derived bioactive compounds and their therapeutic potential in clinical settings.

## 1. Introduction

Cancer cases continue to increase worldwide, and this could be linked both to population growth and the increase in individuals’ average life, as well as to certain bad habits, e.g., smoking, leading to an increase in their occurrence [1]. As reported by GLOBOCAN in 2022, breast cancer (BC) is the leading cause of global cancer occurrence, with an incidence of 2,308,897 cases and mortality of 665,684 subjects. BC aggressiveness is associated with metastasis, relapse and high mortality rates. For every four women with cancer, one suffers from breast cancer and one in six of those die from this type of tumor [2]. Based on clinical characteristics, histology and metastatization phenomena, BC is a heterogeneous and complex disease with specific molecular subtypes (Afifi and Barrero, 2023). Different tumor phenotypes exist, characterized by a complex cellular origin, as well as a heterogeneous progression of the disease, therapeutic response and clinical outcome. Genetic factors, e.g., the *BRCA1* and *BRCA2* genes, may play a key role in giving rise to BC. However, the long-term exposure of breast tissue to estrogen and progesterone is still considered one of the major triggering factors. Indeed, factors which can influence the exposure to estrogen in a woman’s life, e.g., the age of menarche, the first pregnancy or menopause, the total number of pregnancies, as well as hormone replacement therapy, have shown a strong association with the onset of BC [3]. Currently, five important clinical subtypes of BC have been identified, based on their gene expression profiles or response to immunohistochemical (IHC) biomarkers: (i) “normal breast-like” tumors, (ii) “luminal A” [human epidermal growth factor receptor 2 (HER2) negative, progesterone receptor (PR) positive and/or estrogen receptor (ER) positive], (iii) “luminal B” (HER2 positive, PR positive and/or ER positive), (iv) “HER2 overexpressing” (HER2 positive, PR negative, ER negative) and (v) “basal-like” (HER2 negative, PR negative, ER negative, cytokeratin 5/6 positive and/or epidermal growth factor receptor positive). Among these different BC types, Triple-Negative Breast Cancer (TNBC) is the most aggressive phenotype, being only slightly responsive to chemotherapeutic, frequently associated with a poor prognosis and typically characterized by a negative HER2, negative PR and negative ER profile. The diverse BC biological subtypes show different abilities to form metastases in distant organs. Patients with “basal-like” cancer often show lung and central nervous system (CNS) metastases, while in subjects affected by BC luminal subtypes, bone metastases are detected [4]. In MBC, the metastatic event still remains an unclear phenomenon, involving more complex processes, such as genetic and epigenetic mutations, which act to stimulate angiogenesis and tumor–stroma interactions, as well as intravasation through the basement membrane and subsequent extravasation into the target organs [5].

Alongside conventional breast cancer therapies, e.g., breast-conserving surgery (BCS), chemotherapy and radiotherapy, hormonal (endocrine) therapy and immunotherapy, either alone or combined, new promising strategies for BC treatment have recently arisen. In particular, gene therapy, employing vectors to deliver genetic material into target cells to edit genes and change the expression of a gene’s product, is gaining general consensus by the medical community [6]. Moreover, the evolution of next-generation sequencing technologies bodes well for the set-up of precision medicine with tailored treatments in patients suffering from MBC. Specifically, potential therapeutic protocols utilizing immune checkpoint inhibitors (ICIs), epidermal growth factor receptor inhibitor (EGFRi), poly(ADP-ribose) polymerase inhibitor (PARPi), antibody–drug conjugates (ADCs), oncolytic viruses (OVs), glucose transporter-1 inhibitor (GLUT1i) and targeting signaling pathways could become valuable treatment approaches [7].

Nonetheless, MBC’s heterogeneity has highlighted the urgent medical need to develop new effective medicines capable of overcoming drug resistance in this aggressive tumor. In the last few years, much effort has been devoted by the scientific research community to the identification/development of novel compounds with absent, or even reduced, adverse side-effects to be used in oncology for MBC patients’ treatment. In this view, dietary intake of medicinal mushrooms (MMs), an integral part of Traditional Chinese Medicine (TCM) already employed for hundreds of years, displaying specific efficacy against cancer, including BC, has recently aroused great interest [8,9,10,11,12,13]. In fact, the array of bioactive compounds derived from these MMs have been demonstrated to possess modulatory activity effective on peculiar signaling pathways which are misactivated in cancer cells, leading to a recovery of the physiological cellular homeostasis. In particular, the beneficial effects in BC prevention/treatment, due to the anticarcinogenic activity, antimutagenic properties, and onco-immunological and immunomodulatory actions, seem to be linked to the branched polysaccharides (β-glucans) or polysaccharides–protein complexes and the related modulation of the innate and cell-mediated immune response [8]. The power of MMs can also be related to their minerals, amino acids and vitamins (thiamin, riboflavin, ascorbic acid and vitamin D2), which could regulate aberrantly activated signaling pathways in cancer cells and modulate specific molecules involved in cell death and cellular proliferation, as well as their low in fat and high in protein (19-35%), fiber and carbohydrate content [14]. These valuable therapeutic effects have been extensively discussed and demonstrated by preclinical and phase I, II and III clinical trials described in recent review papers, reporting the effect of several MM-derived biomolecules on a variety of human disorders. Undeniably, the latest literature data highlight an extremely wide range of MMs employed in clinical trials, such as *Lentinula edodens*, *Agaricius blazei*, *Agaricius bisporus*, *Pleurotus ostreatus*, *Ganoderma lucidum*, *Agaricius sylvaticus*, *Grifola frondosa*, *Coriolus versicolor*, *Cordyceps sinensis*, and *Hericium erinaceus*. Additionally, more than 250 other MM species have been clearly indicated as useful for human health, with beneficial effects assessed after treatment/supplementation in a wide range of pathological conditions, e.g., diabetes/obesity, ulcerative colitis/Crohn’s diseases, depressive disorders and hepatitis, also including cancer diseases. MMs have been recognized as valuable adjuvant remedies in the difficult management of patients suffering from different cancer types, e.g., colorectal, prostatic, liver, lung, cervical, ovarian, gastric and endometrial tumors, and particularly, for those affected by BC [15,16].

In particular, it has been shown that some MMs, e.g., *Reishi*, *Maitake*, *Agaricus* and *Cordyceps*, are capable of increasing Th1 cytokine production, both in vivo and in vitro, even though these modulatory effects on cytokine expression levels vary accordingly to diverse evaluated experimental models. However, it has emerged that these MMs can downregulate Th2 cytokines, leading to a benefit in cancer treatment [17]. Furthermore, MMs appear to have both a direct effect on the tumor and, parallelly, an immunomodulating systemic effect capable of destroying the tumor. Moreover, the immune-stimulating impact of MMs on T cells, macrophages and NK cells can provide a protective effect against one of the most serious deleterious effects of chemotherapy, i.e., chemotherapeutic myelosuppression, resulting in a better response to treatment [18]. Furthermore, recent experimental data, obtained using an in vivo MBC model, strengthened the protective role of an MM blend, and was able to inhibit the metastatization process, forcing tumor cells to apoptosis; specifically, a striking effect in reducing both cerebellar and lung metastases was revealed, accompanied by an inflammation/oxidative stress decrease in both metastases and the tumor microenvironment, together with QoL and locomotor performance amelioration. Hence, these beneficial pleiotropic outcomes have been hypothesized to be ascribable to either a direct anticancer effect and/or to a secondary/indirect impact of MMs on systemic inflammation/immunomodulation [8,9,19].

Among the bioactive compounds derived from MMs, polysaccharides are the most potent substances in terms of antitumor activity [20]. Overall, MM-derived polysaccharides show a strong antitumor activity against a variety of metastatic cancer cells, particularly when used in combination with conventional treatment protocols, such as chemotherapeutic drugs, surgery, etc. [16]. A multi-tiered in vitro and in vivo study corroborated these data and demonstrated how MMs may mediate an antimetastatic effect even in human MBC, through a possible inhibition of the NFκB signal paralleled by the modulation of Wnt/β-catenin pathways [21]. Furthermore, an in vitro study on MDA-MB-231 breast cancer cells showed the cytotoxic and inhibitory effect played by MM-extracted proteins on tumor cells and migration/metastases formation, also revealing their strong action on apoptosis activation and alteration in the cell cycle in breast cancer cells [22].

Given the high rate of the metastatization process and bad impact of secondary metastases on MBC patients’ survival and QoL, in this review, we summarize the current state of knowledge on five species of MMs, i.e., *Lentinula edodes*, *Agaricus blazei*, *Ganoderma lucidum*, *Grifola frondosa* and *Cordyceps sinensis*, and their derived bioactive compounds, known for their immunomodulatory activities and their capacity to modulate the onset and proliferation of cancer cells, either alone or in mixtures [8,9,19,23,24,25,26,27,28,29]. Particular emphasis has been focused on the description of the intracellular/molecular mechanisms underlying their anticancer action on secondary metastases emerging in distant organs, hence exploring therapeutic potential in clinical settings.

## 2. *Agaricus blazei*

*Agaricus blazei* Murrill, also known as “Cogumelo do Sol” in Brazil, was discovered about 50 years ago [8]. The *A. blazei* medicinal extract has been amply used in the treatment and prevention of cancer, as well as in cardiac diseases, hepatitis, hyperlipidemia, diabetes, dermatitis and atherosclerosis [30,31]. This mushroom has quickly become one of the most popular cultivated MMs in Japan, where it is consumed by about a third of urogenital cancer patients, and it is also one of the most popular MMs in Taiwan [31]. In Japan, about 60.6% of cancer patients who turned to complementary alternative medicine reported using the *A. blazei* mushroom. Additionally, around half a million individuals in Japan have used this MM either to prevent cancer or even as a supplement alongside chemotherapy treatment following the removal of a malignant tumor [23]. Moreover, a phase I clinical study involving repeated administration of *A. blazei* in patients undertaking various cancer therapies, starting from surgery to chemotherapy, has indicated that a supplementary diet including the MM appears to be safe and beneficial [24]. The antitumor activity of beta-glucans from *A. blazei* has been reported in different in vitro and in vivo studies, increasing the clinical interest for these MMs [32]. *Agaricus blazei* Murill is used to treat cancer and other diseases, such as allergy, inflammation and infection, due to its modulatory effects on the innate immune system, probably ascribable to its abundance in immunomodulating molecules such as proteoglycans and highly branched β1,3-/1,6-glucans. *A. blazei* also contains provitamin D2 and agaritine, which can inhibit tumor-induced angiogenesis and induce apoptosis in leukemic cells [33]. Previously published studies also showed the antitumor properties of *A. blazei* in preclinical in vivo models of myeloma, fibrosarcoma, prostate, ovarian and lung cancer, and also in human studies, related to gynecological cancer and leukemia, revealing its ability to increase NK cell activity and parallelly improve QoL [34]. In vitro treatment using *A. blazei* may lead to the strong activation of a caspase-dependent apoptotic mechanism even on pancreatic cancer cells, as demonstrated on HPDE, PCI-35, MIA PaCa-2 and PK-8 cell lines [23]. The potential therapeutic application of estrogenic Erk1/2-activating compounds derived from *A. blazei* has also been demonstrated in in vitro studies on BC using the MCF-7 cell line [35], suggesting a targeted effect in suppressing the proliferation/growth of tumor cells. The antitumor and antimetastatic activity of *A. blazei* is ascribed to its β-d-glycans, which act as immunomodulators, stimulating the production and activation of macrophages, NK and T cells, and plasma cells. These activated lymphocytes lead to increased levels of cytokines (i.e., Interleukin-6 (IL-6) and Tumor Necrosis Factor-alpha (TNF-α)), which, in endothelial cells, result in the regulation of cell adhesion molecules (e.g., E-selectin), able to participate in the mechanism of metastasis formation. *A. blazei*-derived polysaccharides block the activity of TNF-α triggering an alteration in the expression levels of E-selectin, finally resulting in a reduction in the metastatization process [23] (Table 1).

## 3. *Ophiocordyceps sinensis*

*Ophiocordyceps sinensis* (Berk.) (synonym *Cordyceps sinensis*), also called “Dong-Chong-Xia-Cao”, is a parasitic mushroom growing on the larva of the caterpillar, at approximately 4200 m above sea level, amply used in traditional Chinese medicine [36]. In China, *O. sinensis* is widely used as a tonic. However, its applications in oriental medicine range from its simple use in the case of back pain to consumption in the case of cardiovascular, kidney, liver and lung diseases (e.g., from asthma to tuberculosis) [37]. Its action mechanism aims to enhance the response of the immune system and its use, even in combination with conventional therapies, seems to lead to a recovery of physiological oxygen-free radical scavenging, in addition to its antisenescence and antiatherosclerotic effects [38]. It is also regularly used as an adjuvant in cancer therapy, in combination with conventional pharmacological treatment [39]. Many bioactive molecules have been extracted from *O. sinensis*, including nucleosides (e.g., inosine, cytidine, adenine, adenosine, cytosine, uridine, thymidine, guanine, uracil, hypoxanthine, cordycepin and guanosine), Exopolysaccharide fraction (EPSF), acid polysaccharide (APS), CPS-1, CPS-2, sterols (e.g., ergosterol), cordymin, cordycedipeptide A, cordyceamides A and B and tryptophan [40]. An *O. sinensis*-derived polysaccharide, named CSP, is able to induce a strong activation of regulated cell death (RCD) mechanisms such as autophagy and apoptosis, decreasing the proliferation activity of the human colon cancer cell line (HCT116) [41]. CSP stimulates caspase activation, with increased expression levels of caspase 8 and with consequent activation of caspase 3, resulting in stimulation of the apoptotic pathway. Parallelly, this polysaccharide inhibits lysosome formation, leading to the accumulation of autophagolysosomes and activation of the autophagic cell death mechanism. This *O. sinensis*-induced autophagy activation, characterized by the formation of membranous vacuoles, has also been demonstrated in MCF-7 human breast cancer cells, as shown by the increased expression levels of the autophagy marker LC3-II [42]. The antitumor effect of *O. sinensis* has been demonstrated in vitro on the triple-negative breast cancer 4T1 cell line and confirmed in vivo in a mouse breast cancer metastasis model, revealing a potent antimetastatic activity possibly through the down-regulation of metastasis-related cytokines and matrix metalloproteinases expression levels both in serum and the lung [43].

Vascular endothelial growth factor (VEGF) is a key player in the process of angiogenesis, a phenomenon underlying the mechanism of metastasis and tumor growth. The expression levels of this vascular growth factor are actually higher in tumor parenchyma and act as an enhancer of the process of tumor migration and proliferation. *O. sinensis* has been shown to act by blocking the VEGF/VEGFR2 signaling pathway, leading to a reduction in angiogenesis and therefore reducing tumor proliferation and metastases formation [44].

A recent systematic review has also demonstrated that, in a total of 12 randomized clinical trials, including a total of 928 patients, the use of *O. sinensis* as adjuvant treatment in the management of cancer patients led to improved immune function and tumor response rates, with a consequent improvement in patients’ QoL paralleled by the reduction in adverse drug reactions’ incidence, including myelosuppression and thrombocytopenia [25] (Table 1).

## 4. *Ganoderma lucidum*

*Ganoderma lucidum* (commonly known as reishi, varnished conk or ling chih) is an edible mushroom, widely used for decades in Japan and China to promote human longevity. Its therapeutic application ranges from the treatment of allergy, bronchitis and insomnia to use in the management of nephritis, hepatitis and cancer. Its different biological actions can be ascribed to the fruit bodies, mycelia and sporophore extracts. The anticancer effects of *G. lucidum* appear to be related to its biologically active polysaccharides, i.e., branched (1,3)-β-D-glucans. This MM contains up to 83% glucans and a clear correlation has been demonstrated between glucan levels and the oxidizing power of this MM in the fight against free radicals [45]. An in vitro study examined the antioxidant potential of methanolic extracts of *G. lucidum* at different concentrations, revealing its potent free radicals scavenging activity [46]. Another investigation was conducted to assess the effects of *G. lucidum* in the MCF-7 breast cancer cell line, supporting its use as a valuable therapeutic agent against oxidative damage and cancer [47]. The medicinal effects of *G. lucidum*-derived β-glucan polysaccharides seem to be mediated by the complement receptor type 3 (CR3), able to bind these branched polysaccharides. The latter are implicated in the inhibition of tumor growth and metastases formation through the stimulation of the immune system, involving NK cells, T and B lymphocytes and macrophage activation [48]. Parallelly, *G. lucidum* extract seems to be able to modulate expression levels of IFN-γ, TNF-α, IL-1, IL-2 and IL6 and increase the activity of NK cells in late-state lung cancer patients [49]. *G. lucidum*-derived polysaccharides may stimulate IFN-γ (from T cells), TNF-α and IL-1β (from monocyte–macrophages) production in humans, and the increased cytokines levels induced by the fresh fruiting body inhibit human leukemic cell proliferation [50]. Indeed, the *G. lucidum* antitumor effects could be linked to the fruit body, whose extract stimulates the immune response and parallelly inhibits FAS-mediated apoptosis through the activation of the phosphatidylinositol (PI) 3-kinase/AKT pathway and promotes neutrophils’ phagocytosis through the protein kinase C (PKC) and MAPK pathways. Furthermore, the glutathione S-transferase (GST) activity induced by *G. lucidum*-branched polysaccharides showed a chemopreventive effect [51], while the aminopolysaccharide fraction is also able to reduce the ROS production and linked oxidative DNA damage in cancer [52]. *G. lucidum* extracts (GLEs) are able to (i) increase the levels of NK and CD8+T cells in the tumor microenvironment and peripheral immune system, (ii) regulate cell death mechanisms in vitro, by modulating the novel inflammatory cell death mechanisms (i.e., pyroptotic cell death) and (iii) block the metastatic process by inhibiting the angiogenesis, migration, adhesion, invasion and colonization of metastatic BC cells [53]. A beneficial role of *G. lucidum* acting on the apoptosis and invasion activity in the inflammatory breast cancer (IBC) cells has also previously been demonstrated, highlighting a selective viability inhibition, with strong effects on cancer cells only, while the noncancerous mammary epithelial cells remained unaffected [54].

Thus far, a limited number of clinical trials have solely examined the anticancer properties of *G. lucidum*-derived polysaccharides, focusing on the effects of these polysaccharides in combination with other synthetic or natural chemotherapeutic agents, therefore providing novel insights of the potential positive action on the immune system of cancer patients [26] (Table 1).

## 5. *Grifola frondosa*

*Grifola frondosa* mushroom, also known as “Maitake” in Japan, has a long history in complementary and integrative medicine. This MM is uncommon in Europe but grows in North America, China and Japan. This variety reaches 50 cm in width, grows in dense clusters at the bottom of the living hardwoods and has a pleasant smell, the reason why the young and tender basidiomata were classified as edible species in 1821, and are widely appreciated in Japanese cuisine [55]. Recent studies demonstrated that *G. frondosa* extract leads to a down-regulation of inflammation (through a reduction in TNF-α levels) in rodents after captopril treatment. Moreover, *G. frondosa* extract increases IFN activity in patients with invasive bladder cancer, and this effect could probably be ascribed to its high content of ascorbic acid, flavonoids, α-tocopherol residues and β-glucans [56]. Moreover, several glucans, flavonoids and fatty acids derived from *G. frondosa* modulate gene expression and regulate the biological functions of different tumor suppressors in BC cells [57]. In particular, *G. frondosa* may decrease the size of breast and lung cancers, leading to significant symptom improvement or cancer regression in about 70% of BC and around 60% of lung cancer patients [58]. The in vitro and in vivo antitumor effects of *G. frondosa*-derived polysaccharides (GFPs) were demonstrated (i) in two different BC cell lineages, i.e., MCF-7 and MDA-MB-231, and (ii) in MCF-7 tumor-bearing mice; the GFPs-caused cytotoxic effects on cancer cells were observed in terms of increased apoptotic cell death, with enhanced expression levels of the pro-apoptotic markers Bax, caspase 3 and caspase 8 accompanied by reduced levels of the anti-apoptotic molecule Bcl-2, reactive oxygen species overproduction, lactate dehydrogenase release, mitochondrial dysfunction and subsequent reduction in tumor cell viability [59]. Maitake D-Fraction is also able to modify viability in TNBC MDA-MB-231 cell lineage, activating apoptotic cell death and, parallelly, inhibiting different pathways involved in the process of tumor cell metastatization; the expression levels of E-cadherin are also affected by Maitake D-Fraction, increasing cell adhesion between MDA-MB-231 cells and their substrate, and promoting the exposure of β-catenin on the cell membrane. Moreover, the proteoglucan inhibits the tumor cell motility by acting on the rearrangement of the cellular actin component and contemporaneously decreasing the proteolytic activity of MMP-9 and MMP-2; hence these mycotherapy-induced effects trigger a decrease in the invasive capacity of MDA-MB-231 cells [60]. Moreover, *G. frondosa*-derived β-glucans have been shown to enhance therapeutic efficacy and to reduce the myelosuppression and nephrotoxicity of cisplatin in mice, also decreasing spleen- and body-weight alterations [61].

Recently, the use of *G. frondosa* extracts in a phase I/II dose escalation trial on thirty postmenopausal BC patients showed no dose-limiting toxicity and demonstrated immunomodulatory effects measured in peripheral blood [27] (Table 1).

## 6. *Lentinula edodes*

Another popular MM is *Lentinula edodes*, also known as “Shiitake”. This MM is rich in therapeutic polysaccharide macromolecules with demonstrated pharmacological effects: (i) lentinan, a homopolysaccharide with high molecular weight, (ii) KS-2, a peptide–polysaccharide complex and (iii) *L. edodes* mycelia (LEM), a heteroglycan–protein conjugate containing 44% sugars and 24.6% protein [62]. Lentinan is the main antitumor polysaccharide in *L. edodes* fruit bodies, while in the mycelia, different active compounds possess “antitumor” activity, through strengthening of the patients’ immune system [63]. A recent experimental in vivo study aimed at evaluating the hepatoprotective and antioxidant action of *L. edodes* validated its healthy potential as medicine for the liver [64]. Parallelly, several previous in vitro and in vivo studies demonstrated the antitumor activity of LEM extract’s oral administration and how these mycelia extracts can modulate and increase the host immune response [65,66]. Moreover, oral LEM ingestion may reduce inflammation in liver injury in mice, evidencing the anti-inflammatory effects, or inhibit the growth of a subcutaneous B16 melanoma [65]. In particular, the first effect of LEM extract’s ingestion, on the immune system of tumor-bearing hosts, seems to be linked to gut-associated lymphoid tissue (GALT) [65]. Recent in vivo findings in mice evidenced that *L. edodes*-derived polysaccharide extract exerted a remarkable inhibitory effect on the receptor-interacting protein kinase MLKL-RIPK1-RIPK3 necroptosis signaling cascade. This antinecroptotic activity and the consequent anti-inflammatory effects were found to depend on the carbohydrate-rich fraction of the polysaccharides [67]. Moreover, the abovementioned lentinan shows antiviral, anticancer and immunomodulatory activities and exerts its antioxidant, antitumor and immunomodulatory effects on different types of cancer, including BC as well as lung, gastric, liver and brain tumors [28,68]. Moreover, *L. edodes*-derived β-Glucan suppresses BC progression, blocking the inflammatory signaling axes with a direct effect on the AKT/mTOR pathway, resulting in the inhibition of macrophage M2 polarization and activation of autophagic cell death in BC cells [69].

The systematic analysis of over 9500 reported cases of lentinan-associated cancer treatment has demonstrated that this MM can improve patients’ QoL by enhancing the efficacy of conventional cancer treatment, e.g., chemotherapy and radiotherapy, also maintaining the host immune function [28,29] (Table 1).

## 7. MMs and MBC Treatment: Limitations and Challenges

With the aim of including MMs in clinical settings, as beneficial adjuvants in MBC patients’ management, it is crucial to gauge limitations and challenges related to their therapeutic use. Various factors combine to make it demanding regarding decision making for the use of this integrative therapy, therefore leading physicians to adopt great caution. The first hurdle lies in the diverse composition/purity of different MM extracts, which are rarely characterized and standardized, hence affecting their potential interaction with conventional cancer treatments. The combined use of MMs together with conventional cancer therapy was demonstrated to boost the chemotherapeutic agents’ cytotoxic activity (e.g., paclitaxel, oxaliplatin, mercaptopurine, etoposide, doxorubicin, cyclophosphamide, cisplatin, capecitabine and carboplatin), also leading to reduced tumor recurrence and metastases formation. These outcomes were paralleled by a reduction in the expression levels of specific markers of tumor proliferation and invasion, as revealed by the therapeutic beneficial usage in clinical trials [70].

Notably, the desirable effective interactions between MMs and traditional cancer treatments represent a key event in the choice of these integrative remedies for cancer patients. In fact, patients undergoing BC conventional therapy receive a combination of chemotherapy, radiation therapy and/or targeted therapies. The previous literature data demonstrated that MMs are able to balance chemo- and radiotherapy by alleviating adverse side effects, such as anemia, bone marrow suppression, reduced immune resistance and nausea [71]. However, the probability exists that MM-derived biomolecules, interacting with conventional cancer treatments, could either enhance or even inhibit their efficacy, or just cause unexpected adverse effects. Clinical studies reported the beneficial impacts of MM supplementation in cancer therapy, with the main effects spanning from mitigating chemotherapy-related toxicity, also enhancing QoL, to bettering clinical outcomes for oncological patients [72]. In particular, the administration of *A. blazei* extracts, along with conventional chemotherapeutic drugs, triggered a chemotherapy-related side effects decrease, for example, ameliorating patients’ appetite [73]. Parallelly, a preliminary study recorded adverse reactions, e.g., nausea and abdominal symptoms, in cancer patients during their first chemotherapy round, where *L. edodes* mycelia extract was not administered. Interestingly, during the subsequent chemotherapy cycle, when the combined use of LEM extract was adopted, these reported adverse effects were significantly reduced [74]. On the other hand, some studies on BC demonstrate that the use of this extract, employed in combination with conventional chemotherapeutics, neither worsens nor improves the adverse events of pharmacological treatment [75,76], highlighting the need for further in-depth studies investigating the potential synergistic action of mycotherapeutic supplements and standard pharmacological medicines used in combination, with the goal to facilitate the critical care management of cancer patients in clinical practice.

## 8. Future Perspectives

The rearrangement of tumor microenvironment (TME) components, e.g., fibroblasts, immune cells, satellite cells, lymphatic and blood vessels, is one of the main factors involved in tumor development and progression. Tumor cells can gain tumorigenesis, drug resistance and tumor maintenance through a complex signaling network that can directly or indirectly manipulate the function of non-cellular and cellular components, taking advantage of the non-malignant cells. Clinical and experimental studies suggest that a careful analysis of those bidirectional mechanisms of interaction between cancer cells and their surrounding TME is the starting line of scientific progress in the identification of the mechanisms of tumor expansion and invasion [9]. Increasing evidence suggests that chronic inflammation alters the TME through several mechanisms, including tissue remodeling, angiogenesis and pro-inflammatory mediators’ production. Inflammation can be triggered by a variety of different pressures, e.g., carcinogen exposure and obesity, as well as immune dysfunction leading to loss of the oncogene suppressor or tumor activation [77]. Therefore, countless chronic inflammatory conditions predispose healthy cells to neoplastic transformation, and the risk of this transformation into the tumor phenotype is directly proportional to the persistence of the inflammatory state. The persistence of an inflammatory condition can increase the risk of the onset of DNA mutations through the modulation of immuno-stress levels. Both cancer cells and inflammatory actors produce free radicals and soluble mediators, such as chemokines, cytokines and metabolites of arachidonic acid, which will increase reactive species’ production. Ultimately, oxidative stress is one of the mechanisms through which cells respond by activating pathways of programmed cell death or cell survival. In this regard, the obligate suppression of apoptosis and deregulated cell proliferation represents the necessary strategy adopted by cancer cells to support neoplastic progression [78]. Each of the pathways that regulate the proliferative and cell death response in normal cells is deeply altered in most cancers. Considering the key role of inflammation, oxidative stress and proliferation/cell death in the development and progression of breast cancer, and assessing the high invasive capacity of these tumor cells and the great ability to form metastases in organs distant from the primary tumor, it is extremely important to assess the involvement of these pathways in both the TME and metastases in these target organs.

**Figure 1:**
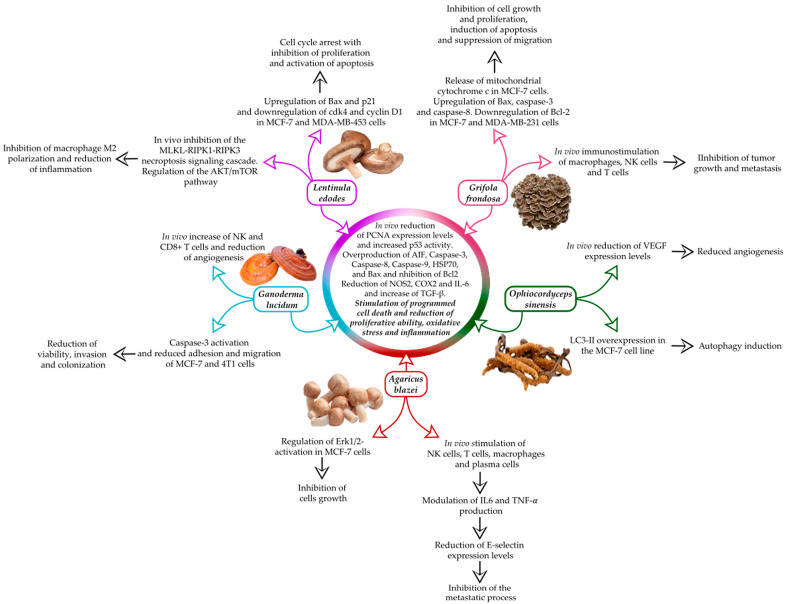
Medicinal mushrooms (MMs) employed as a valuable adjuvant supplement in the treatment of metastatic breast cancer (MBC), and their single and synergic inhibitory effects on the metastatic process.

After the extensive use of conventional and innovative therapies, complementary and integrative medicine (CIM) is currently gaining new value in cancer treatment, given the beneficial effects that this therapy offers to oncological patients, both in terms of QoL amelioration and also in consideration of the improved response to treatment with a reduction in side effects [55]. To evaluate the effects of integrative medicine in the modulation of the metastatic process in MBC, in this review, we summarized the literature data on the most promising MMs, i.e., *Lentinula edodes*, *Grifola frondosa*, *Ganoderma lucidum*, *Ophiocordyceps sinensis* and *Agaricus blazei*, widely used in integrative therapy in Eastern countries, reporting their effect in modulating the immune response, oxidative stress state and proliferation/cell death events in MBC and related metastatic tissue. In addition to the well-established in vivo/in vitro immunomodulatory and anticancer effects, mediated by the use of the single MM on BC [35,42,53,59,69], the recent literature data have extensively proved that a mixture of these five above-reported MMs also showed a strong therapeutic effect on MBC syngeneic tumor-bearing mice, leading to an improved QoL, and also triggering a reduction in the number of lung and CNS metastases; notably, these effects were paralleled by changes in the regulation of inflammatory, oxidative stress and cell death pathways in metastatic MBC cells, in several distant organs from the site of primary tumor onset [8,9,19]. The hypothesis could be that the MM-derived biomolecules may act individually or synergistically, working via two distinct pathways in fighting MBC onset, progression and metastasization. Two different event cascades can be described: (i) an indirect effect occurs by modifying the expression levels of specific cytokines (IFN-γ, TNF-α, IL-1, IL-2, IL-6 and TGF-β) and regulating the inflammatory response by affecting the involvement of adhesion molecules (E-selectins) and angiogenesis (through inhibition of VEGF activity), slowing down the mechanism of metastases formation, and parallelly activating the host immune system through the stimulation of T and NK cells, and (ii) a direct effect on tumor and metastatic cells arises by inhibiting the proliferation of tumor cells, reducing the expression of PCNA and simultaneously increasing the activity of the genome guardian p53, activating mechanisms of programmed cell death (autophagic, apoptotic and pyroptotic cell death) through the activation of specific pro-apoptotic markers, i.e., Apoptosis Inducing Factor (AIF), caspase 3, caspase 8, caspase 9, HSP70 and Bax, and the concomitant inhibition of anti-apoptotic markers (Bcl2) and activation of autophagic molecules (LC3) [8,9,19,42] (Figure 1).

Based on the substantial body of literature documenting the intracellular and immunomodulatory effects observed after MM treatment on primary BC regression, it can be postulated that the same mechanisms could extend to metastatic tumor cells as well. This plausible hypothesis leads to deducing the striking impact of the potential synergistic/additive action of mycotherapeutic supplements and standard pharmacological medicines, used in combination, for the critical care management of MBC patients. Nonetheless, in the imminent future, well-designed clinical trials will be essential to exhaustively assess the efficacy and safety of MMs in MBC patients, also gauging their potential synergistic/additive effect when administered in combination with standard pharmaceutical medicaments. In fact, the majority of the existing evidence pertinent to this topic primarily stems from preclinical studies, highlighting the existence of a critical gap which still needs to be definitively addressed in clinical research for MBC. Hence, by conducting rigorous clinical trials, researchers will have to delve deeper into understanding the benefits and risks associated with MM adjuvant therapy in these patients. It will be crucial to focus on patient outcomes, such as overall survival, progression-free survival, QoL and treatment-related adverse effects. Additionally, considering MBC heterogeneity, further efforts should be devoted to exploring some key points, i.e., the efficacy of specific MM extracts or MM-derived bioactive molecules, the required minimum effective safe dose and the potential synergism, or even additive action, with conventional pharmacological treatments. Yet, it needs to be taken into careful consideration that certain MMs are expensive and some species endangered. Further, the preparation of MM extracts, to be used in clinical settings, requires multiple critical stages, e.g., extraction, fractionation, characterization/standardization, followed by the purification of selected bioactive compounds, hence necessitating significant quantities of bulk-scale materials. Therefore, the availability/sustainability of MMs could pose challenges to their use in a wide clinical scenario. For these reasons, extreme attention must be paid during analytical examination since MM analysis could require “open” methods which can be readily adaptable to identify, with adequate sensitivity, selectivity and specificity, a large number of bioactive molecules/metabolites which could display therapeutic properties against MBC. Chromatographic techniques coupled to mass spectrometry satisfy these needs, also leading to the identification of new unexplored secondary bioactive compounds. In this regard, a recent investigation using ultra-high-performance liquid chromatography combined with triple time-of-flight mass spectrometry (UPLC-TOF/MS/MS) to analyze an MM extract resulted in the discovery of new bioactive compounds with marked anti-inflammatory and anticancer properties [79].

## 9. Conclusions

A wide bulk of the literature data have highlighted the potential therapeutic effects of MMs on BC metastases and the TME, in which an immunomodulatory anti-inflammatory systemic action together with a direct, selective anticancer mechanism exerted a positive pleiotropic effect. In particular, MMs’ preventive and protective effects could affect the TME signaling and, at the same time, target the multifaceted apoptotic pathway. Taken together, these findings corroborate the use of these MMs, alone or in a mixture, as a novel strategy to be used in the field of integrative oncology to improve patients’ QoL with the goal to reduce adverse side effects due to conventional cancer treatments. Thus, although further in-depth studies are necessary to better investigate the clinical setting, turning the use of these five MM varieties into new clinical therapeutic protocols, the present review focused on the use of MMs in the development of clinically relevant therapeutic strategies against MBC. In particular, the growing use of translational research “from bench to bedside” in cancer medicine could allow researchers to overcome challenges which everlastingly hinder medicinal advancements, yielding significant advances in cancer therapeutics and also improvements in the ability to predict the clinical course of a patient’s disease based on individual tumor features. In this view, medicinal mushroom extracts, being natural sources of novel drugs, could be used as an effective adjuvant therapy in the critical management of MBC.

## Figures and Tables

**Table 1:** Summary of presently described MM-derived bioactive compounds and their action/effects on cancer and metastatization process.

Medicinal Mushroom	Bioactive Compound	Mechanism of Action	Outcome
*Lentinula edodes*	Lentinan, *L. edodes* mycelia (LEM) and β-glucans	- Inhibition of MLKL-RIPK1-RIPK3 signaling cascade. - Regulation of AKT/mTOR pathway	- Inhibition of tumor cell proliferation - Patients’ immune system regulation
*Grifola frondosa*	Maitake D-Fraction, ascorbic acid, flavonoids, α-tocopherol residues, β-glucans, flavonoids and fatty acids	- Regulation of TNF-α, IFN-γ, Bax, Bcl-2, caspase-3, caspase-8 and E-cadherin expression levels - Reduction in MMP-9 and MMP-2 proteolytic activity	- Forcing tumor cell death - Immunomodulation
*Ganoderma lucidum*	β-glucan polysaccharides and aminopolysaccharides	- Regulation of IFN-γ, TNF-α, IL-1β, IL-2, IL6 expression levels. - Activation/modulation of phosphatidylinositol (PI) 3-kinase/AKT, protein kinase C (PKC) and MAPK pathways.	- Increased tumor cell death - Immune system activation and regulation - Inhibition of metastatic process by blocking adhesion, migration, invasion, colonization and angiogenesis
*Ophiocordyceps sinensis*	*O. sinensis*-derived polysaccharide (CSP-1 and CSP-2), APS, ergosterol, cordycepin, EPSF	- Regulation of caspase 8, caspase 3, LC3-II expression levels. - Inhibition of VEGF/VEGFR2 signaling pathway.	- Inhibition of BC metastatic process - Immune system activation and regulation
*Agaricus blazei*	Proteoglycans, highly branched β1,3-/1,6-glucans, provitamin D2 and agaritine	- Regulation of IL-6, TNF-α and E-selectin expression levels.	- Suppression of cancer cell proliferation/growth/invasiveness

## Data Availability

Not applicable.
